# Pulsed field ablation for ventricular arrhythmias with pentaspline catheter

**DOI:** 10.3389/fcvm.2025.1631253

**Published:** 2025-07-07

**Authors:** Anna Padisak, Nándor Szegedi, Edit Tanai, Zoltán Salló, Klaudia Vivien Nagy, Péter Perge, Márton Boga, Gábor Orbán, Patrik Tóth, Ferenc Komlósi, Béla Merkely, László Gellér

**Affiliations:** Heart and Vascular Center, Semmelweis University, Budapest, Hungary

**Keywords:** catheter ablation, pulsed field ablation, pentaspline catheter, premature ventricular contraction, ventricular tachycardia, right ventricular outflow tract

## Abstract

**Background:**

Catheter ablation using pulsed-field energy may penetrate deeper into scarred tissue than thermal energies; however, evidence regarding its role in treating ventricular arrhythmias (VAs) is limited. In this prospective study, we report our current experience on pulsed field ablation (PFA) with pentaspline catheter for the treatment of premature ventricular contractions (PVCs) originating from the right ventricular outflow tract (RVOT) and scar-related ventricular tachycardias (VTs).

**Methods:**

Consecutive VA patients who underwent PFA with Farapulse system were enrolled. Seven patients underwent ablation for idiopathic RVOT PVCs, and five patients with structural heart disease underwent ablation for scar-related VTs. The recurrence of arrhythmias was assessed by 24-hour Holter electrocardiography monitoring or implantable cardioverter defibrillator interrogation.

**Results:**

Twelve patients were enrolled, age 51 ± 9 years, nine were men, four had previously failed radiofrequency ablation. Procedural and fluoroscopy times were 53 (41–105) minutes and 8 (4–20) minutes, respectively. The median number of PFA applications was 20 ± 13 in the VT group and 8 (7–8) in the PVC group. Acute procedural success was achieved in 92% (CI 62%–100%) of patients. During a mean follow-up of 100 (97–140) days, freedom from VT was 80% (CI 28%–99%), and a PVC burden <1% was achieved in 71% (CI 29%–96%) of patients.

**Conclusion:**

The ablation of idiopathic RVOT PVCs and scar-related VTs with the pentaspline PFA catheter is feasible, with good acute and mid-term efficacy observed in our cohort. Further research involving larger cohorts and longer follow-up periods is needed to analyze the safety and define the role of PFA in VAs.

## Introduction

Pulsed field ablation (PFA) is a novel ablation method that, unlike conventional techniques such as radiofrequency (RF) and cryoablation, uses a non-thermal energy source. It relies on the application of short, high-energy electrical impulses that result in the irreversible electroporation of cells (1, 2). Its effect is highly selective to cardiomyocytes, lowering the intraprocedural risk of adjacent nerve and esophageal tissue injuries (3, 4). Although PFA was initially developed to perform pulmonary vein isolation, its ability to penetrate deeper into scarred tissue could potentially serve as a solution for overcoming current limitations of RF ablation of ventricular arrhythmias (VAs) (5).

Recently, two studies have been published about focal PFA for the treatment of VAs (6, 7), however, VA ablation using a pentaspline catheter is limited to a few case reports (8, 9).

Our study aims to evaluate the feasibility and efficacy of idiopathic right ventricular outflow tract (RVOT) related premature ventricular contractions (PVCs) and scar-related ventricular tachycardia (VT) ablations using a pentaspline catheter.

## Methods

### Patient population

Consecutive patients undergoing PFA for idiopathic RVOT PVCs or scar-related VTs at Semmelweis University's Heart and Vascular Center in Budapest between October 2023 and September 2024 were enrolled in this study. Ethical approval was waived by the institutional Ethics Committee, and all patients provided written informed consent before the procedure.

Patients with idiopathic RVOT PVCs or scar-related VTs that fulfilled the inclusion criteria were enrolled regardless of previous ablation attempts. Transthoracic echocardiography was used to determine underlying structural heart disease (SHD). PVC patients were all symptomatic before the ablation, with the electrocardiography (ECG) indicating a clear anterior RVOT origin. Pre-procedural 24-hour Holter ECG monitoring was used to assess PVC burden.

The VT group consisted of five patients, with the following underlying conditions: one with arrhythmogenic right ventricular cardiomyopathy, one with Tetralogy of Fallot, one with myocarditis, and two with myocardial infarction. Individual underlying SHDs and scar locations are reported in Table 1. These patients were specifically selected due to the expected presence of extensive scar tissue, presumed to be the primary substrate for the VT, located apically, basally, or laterally, without extensive anterior involvement. All VT procedures were performed in patients with incessant VT, electrical storm, or following failed RF ablation. Each patient had been previously hospitalized due to electrical storms, and all were admitted at the time of ablation with arrhythmic episodes.

**Table 1:** Underlying SHD and scar location in VT patients.

Patient	Underlying SHD	Scar location
Patient 1	ARVC	RVOT, RV posteroseptal
Patient 2	Tetralogy of Fallot	RVOT, RV anterolateral wall
Patient 3	Apical inferior and septal MI	LV apical
Patient 4	IHD, inferior MI	LV anterior, inferior and posterior wall
Patient 5	Myocarditis	RVOT, RV anteroapical

ARVC, arrhythmogenic right ventricular cardiomyopathy; IHD, ischaemic heart disease; LV, left ventricle; MI, myocardial infarction; RV, right ventricle; RVOT, right ventricular outflow tract.

### Ablation system

The FARAPULSE PFA system (Boston Scientific, Marlborough, MA) comprises the FARASTAR generator used to deliver 2 kV biphasic, bipolar pulsed electric field; the FARADRIVE 13Fr steerable sheath; and the FARAWAVE 12Fr, 31 mm, pentaspline single-shot catheter, accommodating four electrodes on each of the five splines.

The FARAWAVE catheter was used in both the fully deployed 31 mm flower configuration and the spherical basket configuration. The CARTO3 mapping system (Biosense Webster, Inc., Diamond Bar, CA) was used for electroanatomical mapping via a PENTARAY catheter (Biosense Webster, Inc., Diamond Bar, CA) in SHD-related VA cases.

### Procedural details

Ablations were performed under general anaesthesia or deep sedation using fentanyl and propofol. For left-sided procedures, systemic heparin was administered with an activated clotting time target of >300 s. Procedures were performed under fluoroscopy guidance and optional intracardiac echocardiography (AcuNav, Siemens Medical Solutions USA, Inc.). Substrate, pace, or activation mapping was used to localize the ideal VT ablation site.

**Figure 1:**
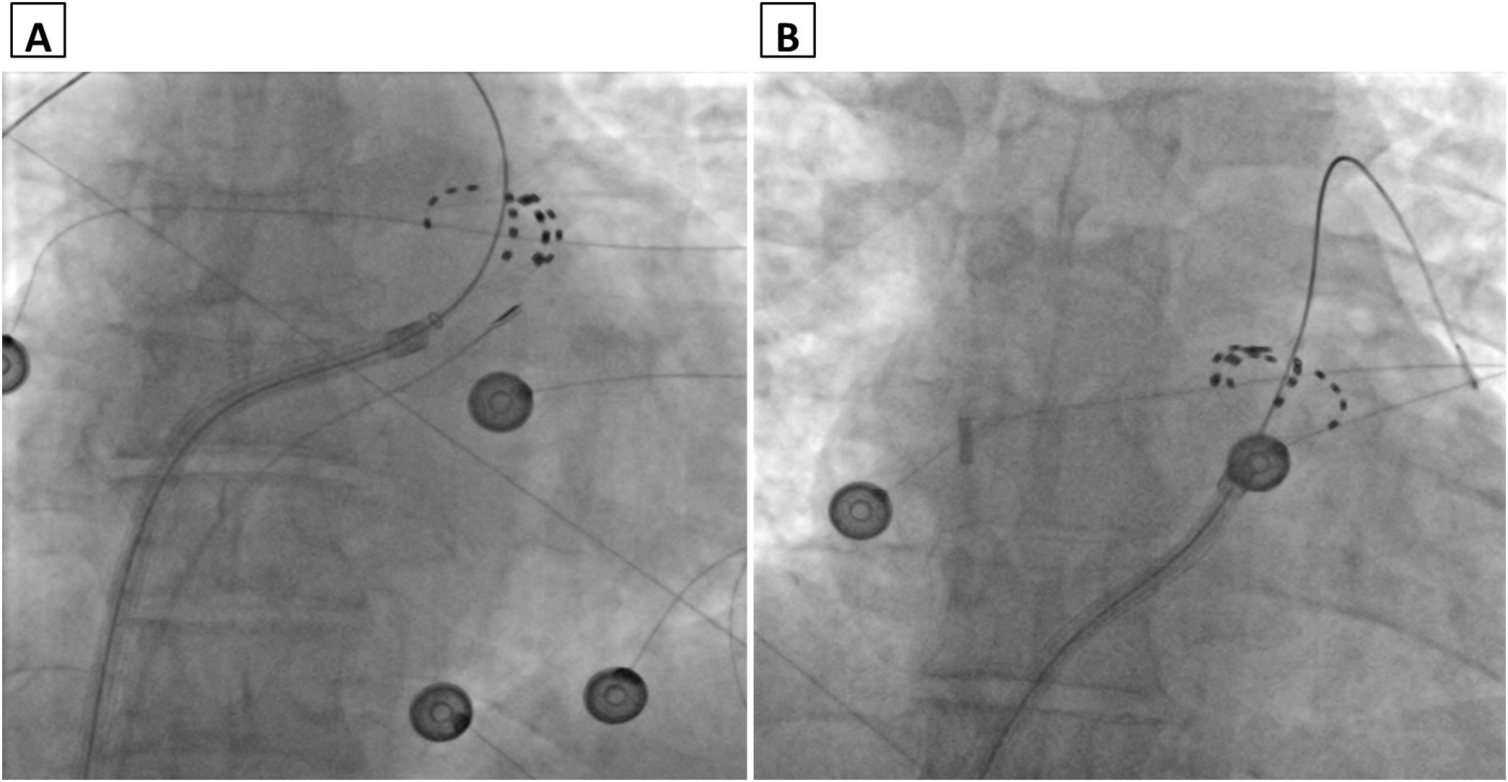
Fluoroscopy image (antero-posterior view) of the pentaspline catheter in “basket” configuration with intracardiac echocardiography catheter in the right ventricular outflow tract **(A)** and right atrium **(B)**.

**Figure 2:**
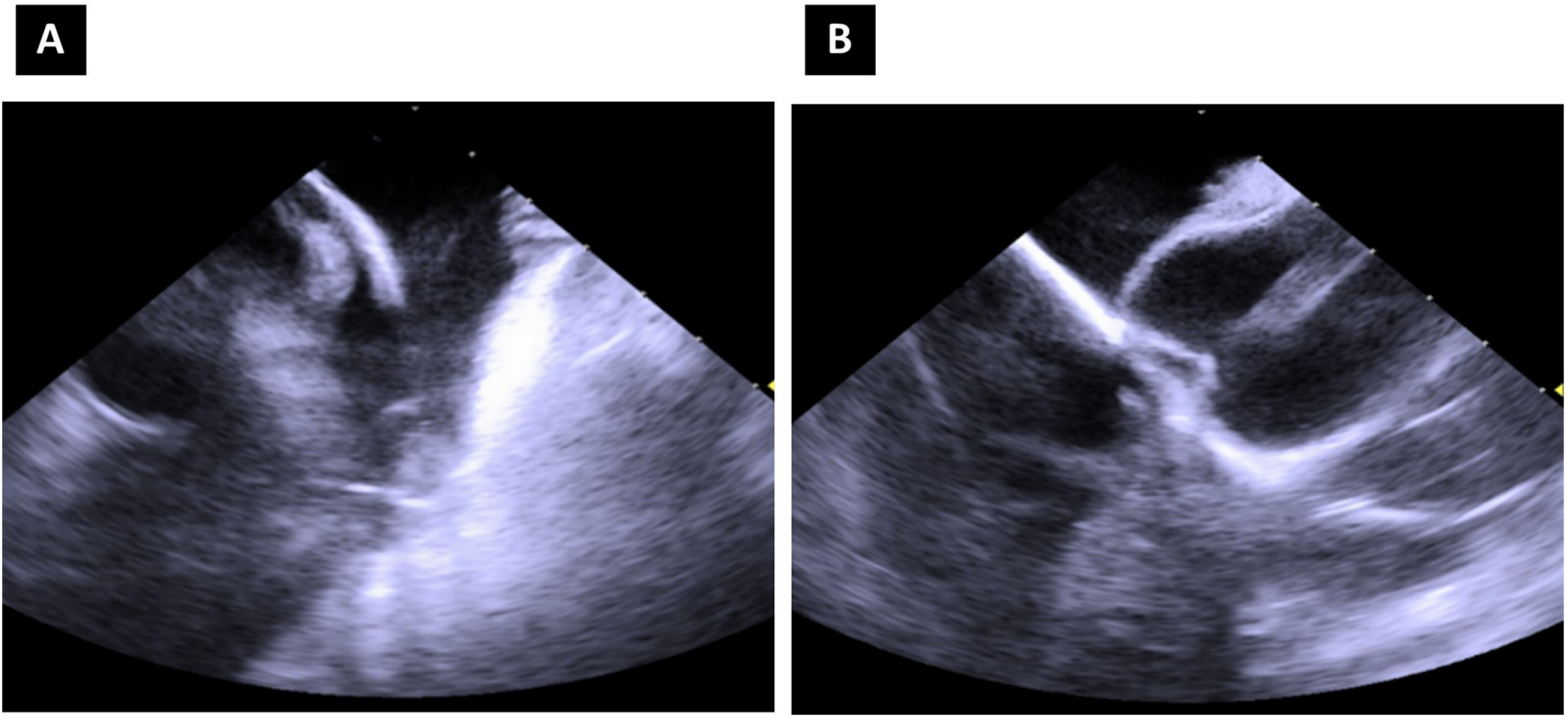
Intracardiac echocardiography image of the pentaspline catheter in “basket” configuration, right ventricular outflow tract view **(A)** and mid-right atrium—“home” view **(B)**.

In patients presenting with PVCs, ablation target sites were localized using 12-lead ECG and earliest activation sites. The FARAWAVE catheter was positioned in the RVOT in basket configuration (Figures 1A,B), and adequate contact was confirmed with intracardiac echocardiography (Figures 2A,B) and by the presence of local electrogram signals recorded by the ablation catheter, which preceded the onset of the QRS complex on the surface ECG. Stable contact was maintained at the desired ablation site through the following steps. First, the sheath was positioned in the right ventricle, and the guidewire was advanced into one of the pulmonary arteries. The ablation catheter was then advanced in a closed position into the RVOT and subsequently deployed in a basket configuration. In this position, the catheter was gently and carefully maneuvered until the earliest ventricular local electrograms were found, usually just below the level of the pulmonary valve. The first set of applications was delivered at this point. After a slight further retraction of the catheter, a second set of applications was performed. All catheter movements were carried out under continuous fluoroscopic guidance. The PVC ablations ended with a 10-minute waiting period to assess the disappearance of PVCs.

**Figure 3:**
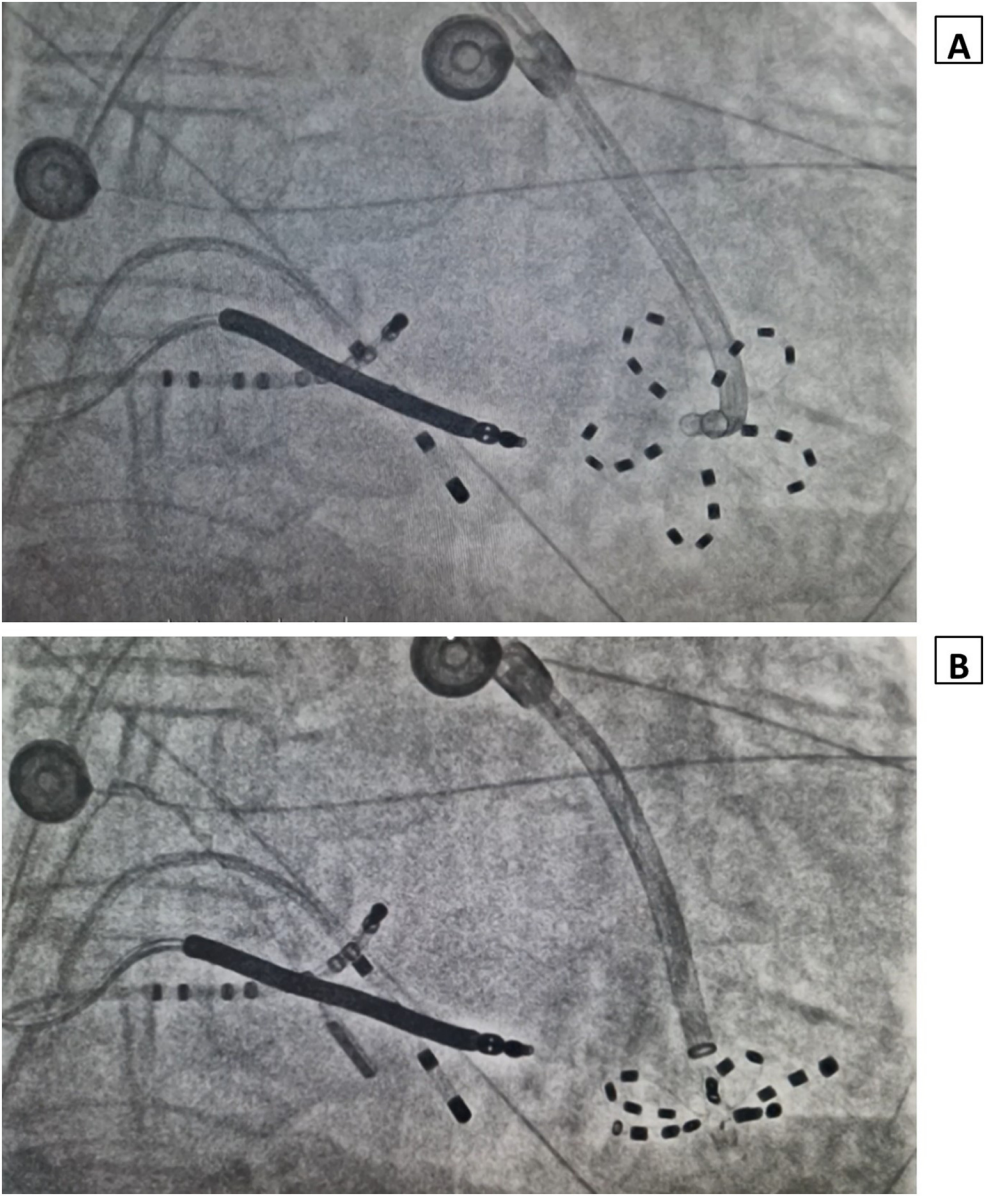
Fluoroscopy image showing the pentaspline catheter deployed in flower configuration at the apico-septal region **(A)** and infero-apical region **(B)** of the left ventricle.

In patients presenting with VTs, the target location for the ablation was selected based on three-dimensional electroanatomical mapping of the substrate with Pentaray catheters. We assessed electrograms for late potentials and signal fragmentation. One patient was already in VT at the beginning of the procedure, while the remaining four patients were in sinus rhythm. In three of these patients, VT was inducible with programmed extra stimulation, and in one patient, VT could not be induced. The left-sided VT ablations were performed as follows. After a transseptal puncture, the sheath was advanced into the left atrium, pointing towards the left ventricle (LV). Then the sheath was advanced into the LV through the mitral valve. The guidewire was then positioned against the ventricular wall, after which the catheter was advanced in a closed position until it reached the cavity of the ventricle. It was then deployed in the fully opened flower configuration (Figures 3A,B), and ablation was performed to homogenize the scarred ventricular tissue. All catheter manipulations were performed under continuous fluoroscopic guidance with utmost caution. Contact was verified through the electrograms recorded by the catheter. A remap with the Pentaray catheter was performed at the end of the procedure, and programmed extra stimulation was used to assess VT inducibility. Throughout the procedures, care was taken to maintain a distance of at least 2–3 centimeters from the conduction system.

All procedures were performed by the same highly experienced operator, and the number of applications was left to the operator's discretion. The number of applications was guided by the operator's prior experience with the catheter and PFA, and the complete disappearance of local electrograms. Nitroglycerin was not routinely administered during the procedures, although it was readily available if needed. Venous punctures were closed using the figure of eight suture. Whenever an arterial puncture was necessary to perform additional RF ablation, the access site was closed using an Angio-Seal device (Terumo Europe N.V., Leuven, Belgium).

In the VT group all patients were receiving amiodarone before the ablation and continued to experience arrhythmic episodes despite this therapy. Amiodarone was maintained after the ablation in all VT patients for at least 3 months. When not otherwise indicated, patients undergoing extensive LV ablations received anticoagulation for 2 months. In the PVC group, antiarrhythmic therapy was discontinued after the acutely successful procedure.

### Success definition

Acute success was defined as the complete elimination of PVCs or the non-inducibility of VTs with programmed extra stimulation pacing. Mid-term success was defined as an overall <1% PVC burden using repeated, high-quality 24-hour Holter ECG monitoring at >3 or >3 months VT-free survival assessed through implantable cardioverter defibrillator (ICD) interrogation. The ICD interrogations were conducted at 3 or 6 months post-ablation or earlier if patients presented with any symptoms.

### Statistical analysis

Continuous variables are expressed as mean ± standard deviation or median and interquartile range, depending on their distribution. For selected variables, both interquartile range and range are reported to better reflect data dispersion. Normality assumptions were tested with the Shapiro–Wilk test. Categorical variables were expressed as counts and percentages, while confidence intervals were calculated using the Clopper-Pearson exact binomial method.

## Results

### Patient population

Twelve patients (75% men) with a median age of 51 ± 9 years undergoing PFA for VA were enrolled in this study. Four patients (33%) have previously undergone at least one unsuccessful RF ablation. In the VT group, two patients had undergone two unsuccessful RF ablations each, while in the PVC group, two patients had undergone one and two unsuccessful RF ablations, respectively. All patients with SHD had ICDs previously implanted. Pre-procedural VT burden was 3 (1–6; range 1–200) based on the number of VT episodes documented by the implantable cardioverter defibrillator and terminated by antitachycardia pacing over the past 100 ± 86 days. 75% of patients were overweight or obese (BMI 29 ± 5). Pre-procedural PVC burden in the PVC group was 24 ± 12% on a 24-hour Holter monitoring. Baseline patient characteristics are reported in Table 2.

**Table 2:** Per group baseline characteristics.

Characteristics	All patients (*N* = 12)	Scar-related VT patients (*N* = 5)	Idiopathic RVOT PVC patients (*N* = 7)
Age (years)	51 ± 9	51 ± 14	50 ± 5
Male sex, *n* (%)	9 (75%)	3 (60%)	6 (86%)
BMI (kg/m^2^)	29 ± 5	28 ± 8	29 ± 3
AHT, *n* (%)	7 (58%)	3 (60%)	4 (57%)
DM, *n* (%)	1 (8%)	1 (20%)	0 (0%)
LVEF (%)	60 ± 7	49 ± 6	60 ± 7
SHD, *n* (%)	5 (42%)	5 (100%)	0 (0%)
ICD, *n* (%)	5 (42%)	5 (100%)	0 (0%)

AHT, arterial hypertension; BMI, body mass index; DM, diabetes mellitus; ICD, implantable cardioverter defibrillator; LVEF, left ventricular ejection fraction; SHD, structural heart disease. Continuous variables are expressed as mean ± standard deviation. Categorical variables are expressed as counts and percentages.

### Procedural characteristics

Eleven procedures were conducted under deep sedation with fentanyl and propofol, and one procedure was conducted under general anaesthesia. A median number of 8 (7–8) pulsed-field (PF) applications were delivered in basket configuration to treat idiopathic RVOT PVCs with procedure and fluoroscopy times of 40 ± 14 and 4 (3–5) minutes, respectively. The dose area product was 636 ± 618 µGym^2^. Procedural times for PVC ablations were relatively short; in two cases, difficulty advancing the guidewire into the pulmonary artery lengthened the procedure.

All patients undergoing VT ablation had ventricular scaring. Targeted VTs were monomorphic and stable enough to allow mapping when present or inducible. The scar region was treated with a median number of 20 ± 13 PF applications, delivered mainly in flower configuration. These procedures lasted 112 ± 36 min, with a fluoroscopy exposure of 22 ± 10 min and a dose area product of 1,855 ± 1,702 µGym^2^. In VT cases, ablation time mainly reflected substrate complexity.

No major procedural complications, coronary vasospasm, new bundle branch blocks, or significant conduction disturbances were observed during the ablations. Additional RF applications were deemed necessary after the PFA applications during one acutely unsuccessful PVC ablation. During this procedure, we observed a reduction in PVC burden but not complete suppression, even after additional RF applications. Procedural characteristics are reported in Table 3.

**Table 3:** Procedural details.

Variable	All patients (*N* = 12)	Scar-related VT patients (*N* = 5)	Idiopathic RVOT PVC patients (*N* = 7)
Procedural time (min)	53 (41–105)	112 ± 36	40 ± 14
Fluoroscopy time (min)	8 (4–20)	22 ± 10	4 (4–5)
Fluoroscopy dose (µGym^2^)	644 (436–1,460)	1,855 ± 1,702	636 ± 618
PFA applications, *n*	8 (8–12)	20 ± 13	8 (7–8)
Acute success, *n* (%)	11 (92%)	5 (100%)	6 (86%)
Mid-term success, *n* (%)	9 (75%)	4 (80%)	5 (71%)

PFA, pulsed field ablation. Continuous variables are expressed as mean ± standard deviation or median (interquartile range), depending on their distribution. Categorical variables are expressed as counts and percentages.

### Clinical outcomes

The acute success rate was 86% (CI 42%–100%) and 100% (CI 48%–100%) in the PVC and in the VT group, respectively. The median follow-up duration in the PVC group was 106 (98–140) days, with a mid-term success rate of 71% (CI 29%–96%). The mid-term success rate was 80% (CI 28%–99%) in the VT group at a median follow-up time of 106 ± 58 days.

## Discussion

To the best of our knowledge, this study represents the first evaluation of PFA with a pentaspline catheter for idiopathic RVOT PVCs and scar-related VTs in a larger case series.

Irreversible electroporation was first approved for the treatment of tumours and was only later proposed as an ablative strategy for cardiac arrhythmias (10). Over the past years, it has proven to be efficient in the treatment of atrial fibrillation (11, 12).

Recent studies have shown that PF energy can penetrate deeper into scarred tissue than RF (5, 13). These findings portray PFA as a promising option for treating VAs, where scarred tissue may disable deep lesion creation with other energy sources.

PF energy selectively disrupts cardiomyocyte membranes without causing thermal damage to surrounding tissues (13). Nevertheless, when using large-footprint catheters, the broader ablation area associated with PF energy raises the likelihood of conduction system damage (14). In our study, this was avoided by maintaining a minimum distance of 2–3 cm from the conduction system, and therefore, no bundle branch blocks or significant conduction abnormalities were detected (15, 16).

In the VT group, the FARAWAVE single-shot PFA catheter, deployed in the flower configuration, allowed for rapid and effective homogenisation of large, scarred areas, resulting in successful VT suppression. In the PVC group, the partially deployed basket configuration could easily be adapted to the size of the distal RVOT, facilitating efficient PF energy application. Given the importance of catheter-tissue contact in lesion formation, ICE can be a valuable tool during PVC ablation for creating durable lesions and improving outcomes (17).

This pivotal study aligns with other low-volume research, demonstrating that PFA for ventricular arrhythmias may be effective, even in cases where previous RF ablation failed (6–9, 13, 18–22).

## Study limitations

Some limitations in this study should be addressed. Our findings cannot be extrapolated to other PFA systems. Furthermore, the small sample size of our study may have influenced the statistical power of our observations, which does not allow us to conclude on the safety and long-term efficacy of the procedure. Moreover, patients with VT but no SHD were excluded from this study.

## Conclusion

The ablation of idiopathic RVOT PVCs and scar-related VTs with a pentaspline PFA catheter is feasible and seems effective, with a good acute and mid-term success rate. Further research involving larger cohorts and longer follow-up periods needs to be conducted to evaluate the safety and long-term efficacy of PFA in VAs.

## Data Availability

The datasets presented in this article are not readily available because data will be made available upon reasonable request. Requests to access the datasets should be directed to laszlo.geller@gmail.com.
